# Mutations in the Voltage Sensors of Domains I and II of Na_v_1.5 that are Associated with Arrhythmias and Dilated Cardiomyopathy Generate Gating Pore Currents

**DOI:** 10.3389/fphar.2015.00301

**Published:** 2015-12-24

**Authors:** Adrien Moreau, Pascal Gosselin-Badaroudine, Mohamed Boutjdir, Mohamed Chahine

**Affiliations:** ^1^Centre de Recherche de l’Institut Universitaire en Santé Mentale de Québec, Quebec CityQC, Canada; ^2^Cardiovascular Research Program, VA New York Harbor Healthcare System, BrooklynNY, USA; ^3^Department of Medicine, Université Laval, Quebec CityQC, Canada

**Keywords:** *SCN5A*, gating pore current, arrhythmias, dilated cardiomyopathy, voltage sensor

## Abstract

Voltage gated sodium channels (Na_v_) are transmembrane proteins responsible for action potential initiation. Mutations mainly located in the voltage sensor domain (VSD) of Na_v_1.5, the cardiac sodium channel, have been associated with the development of arrhythmias combined with dilated cardiomyopathy. Gating pore currents have been observed with three unrelated mutations associated with similar clinical phenotypes. However, gating pores have never been associated with mutations outside the first domain of Na_v_1.5. The aim of this study was to explore the possibility that gating pore currents might be caused by the Na_v_1.5 R225P and R814W mutations (R3, S4 in DI and DII, respectively), which are associated with rhythm disturbances and dilated cardiomyopathy. Na_v_1.5 WT and mutant channels were transiently expressed in tsA201 cells. The biophysical properties of the alpha pore currents and the presence of gating pore currents were investigated using the patch-clamp technique. We confirmed the previously reported gain of function of the alpha pores of the mutant channels, which mainly consisted of increased window currents mostly caused by shifts in the voltage dependence of activation. We also observed gating pore currents associated with the R225P and R814W mutations. This novel permeation pathway was open under depolarized conditions and remained temporarily open at hyperpolarized potentials after depolarization periods. Gating pore currents could represent a molecular basis for the development of uncommon electrical abnormalities and changes in cardiac morphology. We propose that this biophysical defect be routinely evaluated in the case of Na_v_1.5 mutations on the VSD.

## Introduction

Voltage-gated sodium channels (Na_v_) are transmembrane proteins responsible for the initiation and propagation of action potentials in many excitable cells. Cardiac action potentials are initiated by Na_v_1.5, the main Na_v_ isoform in the heart. This large 2016-amino-acid protein is encoded by the *SCN5A* gene and is composed of 24 transmembrane segments organized in four homologous domains (Gellens et al., 1992). The assembly of the S5–S6 segments of each domain forms the alpha pore, which is responsible for the selective permeation of Na^+^ ions that underlies the physiological functions of the channel. The S1–S4 segments of each domain make up the voltage sensor domain (VSD), which modulates the activity of the channel through conformational changes induced by variations in the transmembrane voltage. Due to the crucial role of these channels in the generation and regulation of the electrical signals, dysfunctions result in pure arrhythmic phenotypes. For instance, Na_v_1.5 dysfunctions result in cardiac pathologies such as Brugada syndrome, type 3 Long QT syndrome, sick sinus syndrome, and cardiac conduction disorders with no structural heart disease (Amin et al., 2010).

**FIGURE 1:**
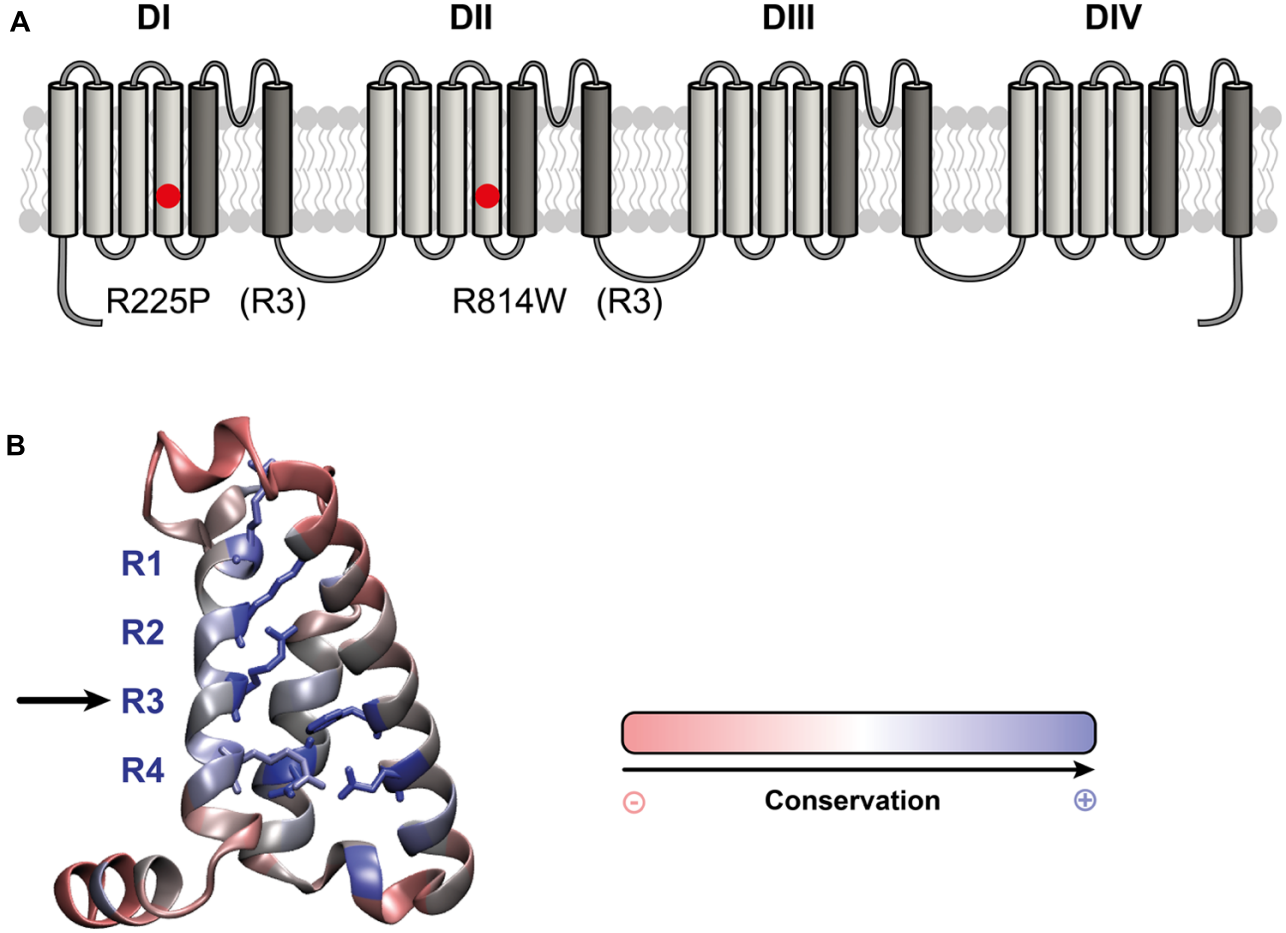
**Location of the R225P and R814W mutations that target highly conserved residues in VSDs. (A)** 2D structure of Na_v_1.5 channels showing the 24 TM segments organized into 4 domains (DI-DIV), each containing 6 TM. The S1-S4 segments of each domain form the VSD (light gray) while the assembly of the S5–S6 segments (dark gray) form the pore of the channel. The locations of the R225P and R814W mutations are indicated by red circles. **(B)** 3D crystal structure of the VSD of Na_v_Ab channel showing the residues that are conserved in all VSDs. These residues were determined by calculating the Shannon entropy at every position for the 6,652 VSD sequence alignments reported by (Palovcak et al., 2014). Highly conserved residues are in blue while less conserved residues are in red. The S1 segment was removed for purposes of clarity. The positively charged residues of the S4 segment (left) are highly conserved as are the residues forming the GCTCs of the S2 and S3 segments (right and middle respectively).

Na_v_1.5 mutations have recently been linked to the development of atypical phenotypes associating cardiac arrhythmias and dilated cardiomyopathy (Bezzina et al., 2003; McNair et al., 2011; Laurent et al., 2012; Mann et al., 2012; Nair et al., 2012; Beckermann et al., 2014; Gosselin-Badaroudine et al., 2014; Moreau et al., 2014a, 2015). The exact pathological mechanism is not completely understood. While the clinical phenotypes are similar, some mutations result in a gain of channel function, some in a loss of channel function, and some have no effect on normal channel function (Bezzina et al., 2003; Gosselin-Badaroudine et al., 2012b, 2014; Laurent et al., 2012; Mann et al., 2012; Nair et al., 2012; Moreau et al., 2014a, 2015). Two Na_v_1.5 mutations located at homologous positions on the S4 segments of DI and DII (R225P and R814W; **Figure 1**) have been characterized (Nguyen et al., 2008; Beckermann et al., 2014). These mutations were found in patients whose phenotypes feature complex arrhythmias associated with dilated cardiomyopathy. Atrial flutter and non-sustained ventricular tachycardia were reported for the patient carrying the R814W mutation (Olson et al., 2005). The patient Na_v_1.5 R225P carrier experienced atrial and ventricular tachycardia, 2:1 atrio-ventricular block, ventricular, atrial ectopy and prolonged QTc (Beckermann et al., 2014). All carriers also demonstrate dilated cardiomyopathy with reduced ventricular ejection fraction (Olson et al., 2005; Beckermann et al., 2014). There is currently no consensus on the exact pathological mechanism linking morphological changes in the heart with a channel typically associated with pure arrhythmic phenotypes.

Interestingly, most of the reported mutations (9 of 13) are located on the positively charged S4 segment of the VSD (McNair et al., 2011; Gosselin-Badaroudine et al., 2012b, 2014; Moreau et al., 2014a, 2015). This segment drives the conformational changes of the VSD in response to changes in voltage (Moreau et al., 2014b). In the course of its movement, the positively charged amino acids of the S4 segment sequentially interact with their negatively charged counterparts on the S1–S3 segments (Delemotte et al., 2011). This arrangement of negatively charged amino acids on the S1–S3 segments is called the gating charge transfer center (GCTC; Tao et al., 2010). Mutations of the positively charged amino acids of the S4 segment disrupt interactions between the S4 segment and the GCTC, resulting in the creation of a new permeation pathway that passes directly through the usually non-conductive VSD. This new permeation pathway is called the gating pore (or omega pore; Sokolov et al., 2005, 2007; Tombola et al., 2005; Struyk and Cannon, 2007; Gosselin-Badaroudine et al., 2012a; Moreau et al., 2014a,b, 2015).

We hypothesized that gating pores might be a common pathological mechanism of several VSD mutations linked to cardiac arrhythmias and dilatation (Gosselin-Badaroudine et al., 2014; Moreau et al., 2014a, 2015). We previously showed that the R219H, R222Q, and R225W mutations in DI of the cardiac Na^+^ channel cause gating pore currents (Gosselin-Badaroudine et al., 2012b; Moreau et al., 2015).

We used a heterologous expression system (tsA201 cells) to study the R225P and R814W mutations (R3, S4 in DI and DII, respectively; **Figure 1**; Nguyen et al., 2008; Beckermann et al., 2014) to determine whether gating pores are present. The characterization of both mutations confirmed the previously observed biophysical defects. We also showed that mutations in DI and DII both result in gating pore currents.

We propose that this permeation pathway may participate in the pathological mechanism. We further propose that this pathway should be routinely evaluated in future studies on *SCN5A* mutations in the VSD of the channel that are associated with arrhythmias and dilated cardiomyopathy.

## Materials and Methods

### Cell Culture

TsA201 cells, which are modified HEK-293 (human embryonic kidney) cells stably transfected with simian virus 40 large T antigen, were used for patch clamp experiments (Moreau et al., 2012). The cells were cultured in high-glucose DMEM supplemented with 10% fetal bovine serum (FBS), 2 mM L-glutamine, 100 U/ml of penicillin G, and 10 mg/ml of streptomycin (GIBCO- BRL Life Technologies, Canada) at 37°C in a 5% CO_2_ humidified atmosphere. The cells were transiently transfected with WT or mutant Na_v_1.5 cDNA (5 μg) using the calcium phosphate method as previously described (Deschenes et al., 2000). The Na_v_1.5 α-subunit was co-transfected with the human β1-subunit. Briefly, 5 μg of each cDNA was mixed with 500 μl of a 250 mM CaCl_2_ solution to which 500 μl of HeBS2x (0.28 M NaCl, 0.05 M HEPES, 1.5 M Na_2_HPO_4_, pH 7.05) was added. Given the size of plasmids and genes inserted, we transfected a ratio of 2 β1 subunits for each alpha subunits.

### Patch-Clamp Recordings

Macroscopic Na^+^ currents (alpha currents) were recorded at room temperature 48–72 h after transfection using the whole-cell configuration of the patch-clamp technique, as previously described (Huang et al., 2011). The liquid junction potential between the patch pipette and the bath solution were not corrected. P/4 leak subtraction was only used for the alpha pore current recordings. For the gating pore current recordings, linear leak subtraction at hyperpolarized voltages was performed off-line to eliminate the inherent non-specific leak. Currents were recorded at a sampling rate of 83.33 kHz and were low pass filtered at 5 kHz. All currents were recorded using a pipette solution composed of (in mM): 135 CsF, 5 NaCl, 10 EGTA, and 10 HEPES (pH 7.4). The bath solution was composed of (in mM): 115 NMDG, 20 NaCl, 2 CsCl, 2 Ca(OH)_2_, 10 HEPES, and 10 TEA-Cl (pH 7.4). TEA-Cl was used to block endogenous potassium currents. Niflumic acid (1 mM) freshly prepared in 100% ethanol was added to the bath solution to block endogenous chloride channels before recording the gating pore currents. When indicated, 10 μM TTX (Latoxan, France) was added to the bath solution to block alpha pore currents.

### Molecular Modeling

We used the procedure described by Moreau and coworkers to generate models for the WT and mutated VSDs for both DI and DII (Moreau et al., 2015). Briefly, the high-resolution of the pre-activated Na_v_Ab structure released in Payandeh et al. (2011) in which all VSDs were considered to be in the activated position was used as a template (accession code 3RVY). Standard MODELER routines (Eswar et al., 2006) were used to generate comparative models of the DI and DII VSDs of the Na_v_1.5 channels. Homology models in which the R225 and R814 residues were in the GCTC were generated using a procedure described previously (Wood et al., 2012; Moreau et al., 2015). Since the activation/deactivation mechanism involves a sliding S4 helix relative to a static S1-S3 bundle with discrete steps during which the positive residues sequentially interact with the GCTC (Delemotte et al., 2011; Gosselin-Badaroudine et al., 2012a; Henrion et al., 2012; Jensen et al., 2012; Moreau et al., 2015). The alignment of the S4 segments used for homology modeling was shifted by three amino acids toward the C-terminus in order to align R225 and R814 to the residue designated as K4 by Payandeh et al. (2011). These alignments correspond to a meta-stable state previously named β state (Delemotte et al., 2011). In this state, R225 and R814 interact with the GCTC.

The homology models were then inserted in a POPC (1-palmitoyl-2-oleoyl-sn-glycero-3-phosphocholine) bilayer and were equilibrated under normal constant temperature and pressure conditions (298 K, 1 atm.) in a 150 mM NaCl solution. The lipid tails were melted during the first nanosecond while restraining the positions of the protein, the lipid head groups, the water molecules, and the ions at their initial positions. Afterward, only the protein was restrained for 2 ns. The restraints were relaxed progressively for 6 ns. Lastly, a 100-ns unrestrained MD simulation of the entire channel was conducted, enabling the system to relax. The MD simulations were carried out using the NAMD2 program. Langevin dynamics were applied to keep the temperature (300 K) fixed. The equations of motion were integrated using a multiple time-step algorithm. Short- and long-range forces were calculated every 1 and 2 time-steps, respectively. The time steps were 2.0-fs long. The simulation used the CHARMM22-CMAP force field with torsional cross-terms for the protein and CHARMM36 for the phospholipids.

Mutant VSDs were created by introducing the mutation into the equilibrated WT VSD using MODELER. They were equilibrated using the same procedure. The simulations were performed on the Calcul Québec/Compute Canada Colosse supercomputer. The operation of the supercomputer is funded by the Canada Foundation for Innovation (CFI), NanoQuébec, RMGA, and the Fonds de recherche du Québec – Nature et technologies (FRQ-NT).

### Data Analysis and Statistics

The electrophysiological data were analyzed using Clampfit (pCLAMP v10.0, Molecular Devices) and custom MATLAB programs (The MathWorks, Inc.). Data are expressed as mean ± SEM (standard error of the mean). When indicated, an ANOVA with a Dunnett *post hoc* test or a *t*-test was performed. Differences were considered significant at a ^∗^*P* < 0.05, ^∗∗^*P* < 0.01, or ^∗∗∗^*P* < 0.001.

## Results

### The R225P and R814W Mutations Cause a Gain of Function of the Alpha Current

**FIGURE 2:**
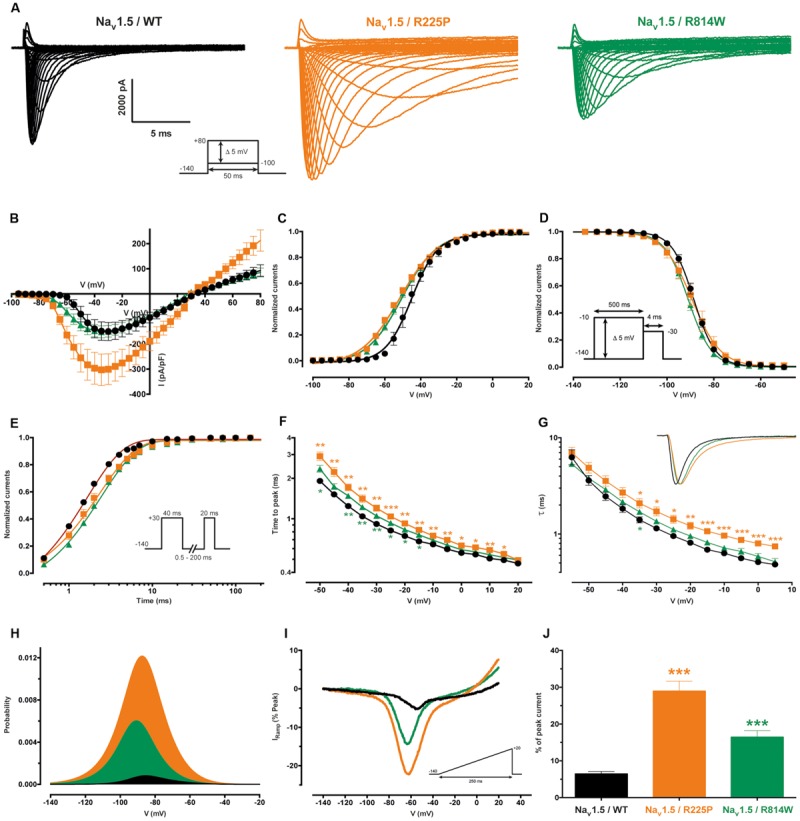
**Biophysical characterization of the Na_v_1.5 mutant channels.** The values for the Na_v_1.5 WT channel are indicated by black symbols, those for the R225P mutant channel by orange symbols, and those for the R814W mutant channel by green symbols. **(A)** Representative whole-cell current traces of the WT and mutant channels (see protocol in inset). **(B)** Current density-voltage (I–V) relationships of the WT and mutant channels. **(C)** Voltage-dependence of steady-state activation of the WT and mutant channels. Activation curves were generated using a standard Boltzmann distribution [*G*(*V*)/*G*max = 1/(1+exp(-(*V*-*V*_1/2_)/*k*))] and gave the V_1/2_ and *k* values listed in **Table 1**. **(D)** Steady state inactivation of the WT and mutant channels (see protocol in inset). The recorded inactivation values were fitted to a standard Boltzmann equation [*I*(*V*)/*I*max = 1/(1+exp((*V*-*V*_1/2_)/*k*))+C] and gave the values listed in **Table 1**. **(E)** Recovery from fast inactivation values were obtained using a two-pulse protocol at +30 mV to obtain maximal activation (see protocol in inset). The time constants listed in **Table 1** were obtained using a single-exponential function: (A_fast_ x (1 – exp(-t/τ_fast_)) + C). **(F)** The times to peak of the WT and mutant channels were used to evaluate activation kinetics. The times to peak were measured from the same current traces used to construct the I-V relationship **(A)**. **(G)** The time constants of fast inactivation decay were plotted as a function of voltage for the WT and mutant channels. The time constants were obtained using a simple-exponential function: (A x (exp(-t/τ) + C). Normalized raw data shown in the inset illustrate the current decay kinetics. **(H)** The overlap between activation and inactivation defines the window current. The predicted window current was obtained using the following equation: (1/(1 + exp((V_1/2activation_ – V)/k_activation_)) x ((1 – C)/(1 + exp((V – V_1/2inactivation_)/k_inactivation_)) + C). **(I)** Ramp protocols (see protocol in inset) were imposed (0.64 mV/ms) to study the window current. Ramp current traces were normalized to the alpha peak current. **(J)** Histogram showing the peak window current normalized to the alpha peak current (% of peak current). ^∗^Difference vs. Na_v_1.5/WT using ANOVA (^∗∗∗^*P* < 0.001).

As previously described (Nguyen et al., 2008; Beckermann et al., 2014), the R225P and R814W mutations both induce a gain of channel function. All experiments are presented in **Figure 2** while in **Table 1** all values and number of independent experiments are listed (**Figure 2** and **Table 1**). However, the defects induced by the R814W mutation were less pronounced than that of the R225P mutation. Both mutations increased the opening probability at hyperpolarized voltages [left shift of their half voltage (V_1/2_) of activation, increased slope parameter]. R225P mutant channels displayed a larger slope factor for their steady-state inactivation curve while R814W mutant channels displayed a left shift in the V_1/2_ of inactivation. Both mutations produced a slower recovery from inactivation, an increase in time to peak, and a slow current decay. These alterations in the biophysical properties of the channel resulted in an increase in channel function through an increase in the window current as well as an increase in channel availability as evidenced by the calculated open probability and the ramp currents (**Figure 2**).

**Table 1:** Biophysical parameters of Na_v_1.5/WT, R225P, and R814W.

	WT (*n* = 7)	R225P (*n* = 8)	R814W (*n* = 9)
**Peak current (pA/pF)**	-154.4 ± 37.4	-303.8 ± 62.5*	-151.8 ± 23.8
**Activation**			
V_1/2_ (mV)	-44.1 ± 1.9	-50.6 ± 1.0**	-49.6 ± 1.3*
k (mV)	-6.9 ± 0.6	-10.1 ± 0.3***	-9.4 ± 0.3***
**Inactivation**			
V_1/2_ (mV)	-88.6 ± 0.7	-89.2 ± 1.9	-91.0 ± 0.8*
k (mV)	4.6 ± 0.2	5.3 ± 0.2*	4.9 ± 0.2
**Recovery from inactivation**			
τ (ms)	2.2 ± 0.1	3.0 ± 0.2**	3.2 ± 0.2***
**Peak ramp current (% peak current)**	6.4 ± 0.7	29.0 ± 2.7***	16.5 ± 1.7**

*V_*1/2*_, Mid point for activation or inactivation.*

*K, Slope factor for activation or inactivation.*

*^∗^Difference vs. Na_v_1.5/WT using ANOVA (^∗^*P* < 0.05, ^∗∗^*P* < 0.01, ^∗∗∗^*P* < 0.001).*

### The R225P and R814W Mutations both Create Gating Pores that are Activated at Depolarized Potentials

**FIGURE 3:**
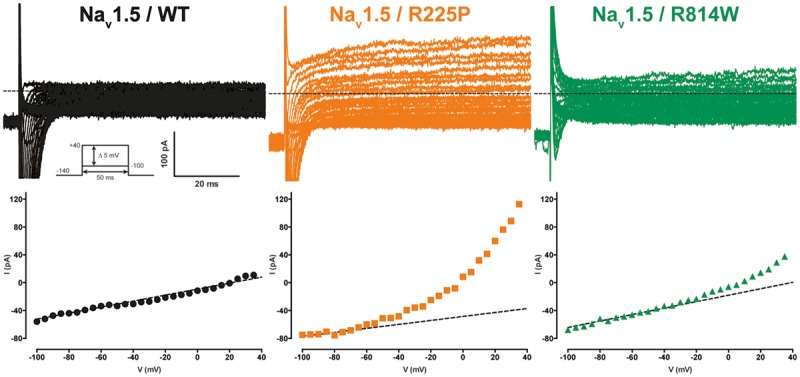
**Gating pore currents of the Na_v_1.5 R225P and R814W mutant channels.** Gating pore currents were recorded in absence of TTX. The cells were held at -100 mV and currents were recorded using a voltage-step protocol from -100 to +40 mV in 5 mV increments. The top panels show examples of raw traces of gating pore currents. The currents are plotted as a function of voltage in the bottom panels. Linear non-specific leaks are indicated by dotted lines.

**FIGURE 4:**
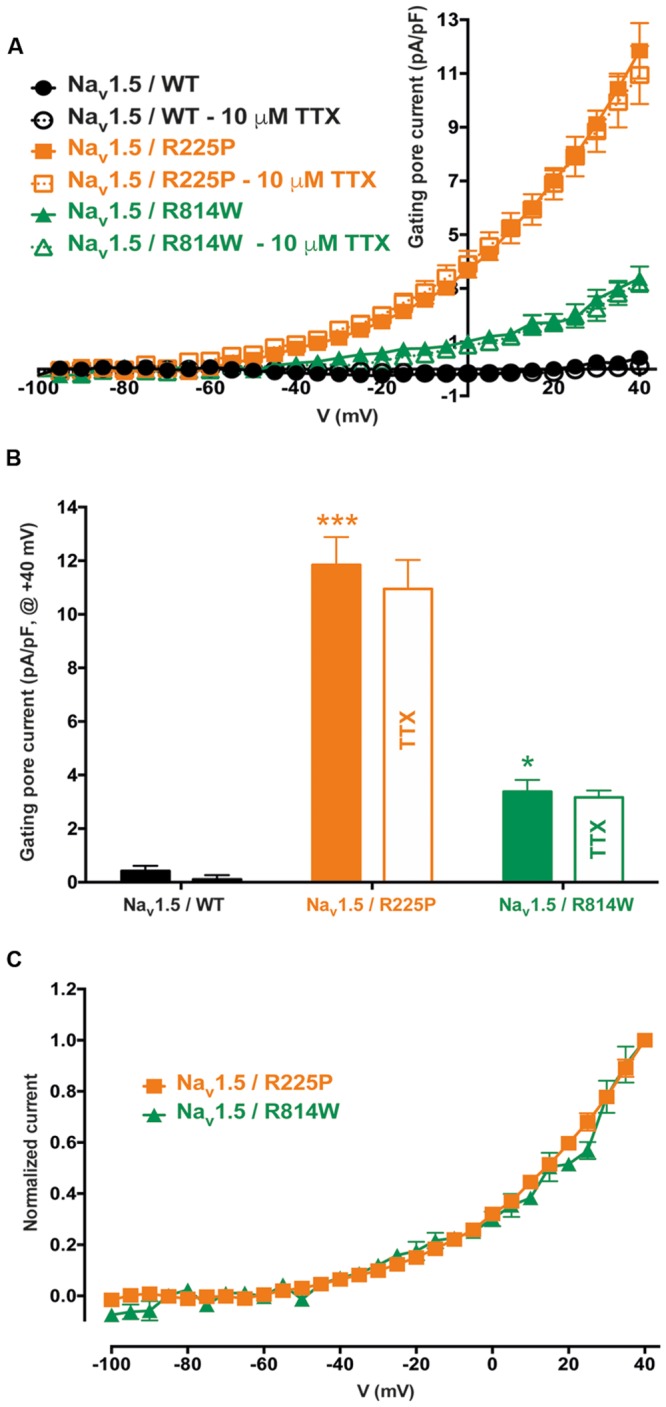
**Voltage dependence of gating pore currents. (A)** Current density-voltage relationships of gating pore currents recorded for the WT, R225P, and R814W channels are shown as filled symbols in the absence of TTX (*n* = 5 for R225P, *n* = 4 for R814W, and *n* = 4 for WT channels) and as empty symbols in the presence of 10 μM TTX (*n* = 6 for R225P, *n* = 3 for R814W, and *n* = 5 for WT channels). **(B)** Histograms summarizing the gating pore current densities at +40 mV for the WT, R225P, and R814W channels (0.4 ± 0.2 pA/pF, 11.8 ± 1.0 pA/pF, and 3.4 ± 0.4 pA/pF, respectively). No differences were observed with or without TTX for any of the conditions. **(C)** Normalized gating pore currents (I/I_max_) exhibiting similar voltage dependence for both the R225P and R814W mutant channels. ^∗^Difference vs. Na_v_1.5/WT (^∗∗∗^*P* < 0.001).

Gating pores are an alternative permeation pathway created in the usually non-conductive VSDs of voltage-gated ion channels by the disruption of interactions between the S4 segment and the GCTC (Moreau et al., 2014a,b, 2015). Due to the nature and location of the R225P and R814W mutations (R3 and S4 in DI and DII, respectively), we hypothesized that they might generate gating pore currents. Unlike WT channels, gating pore currents were induced by the R225P and R814W mutations when 80-ms voltage steps ranging from -100 to +40 mV in 5 mV increments were applied. Because P/4 leak subtraction was not used to record the gating pore currents, linear leak subtraction at hyperpolarized voltages, when gating pores should be closed, was performed off-line to eliminate the non-specific leak inherent to the patch-clamp method (**Figure 3**, bottom panels). The gating pore currents created by the R225P mutation were larger than those created by the R814W mutation [at +40 mV: 11.8 ± 1.0 pA/pF for R225P (*n* = 5), 3.4 ± 0.4 pA/pF for R814W (*n* = 4), and 0.4 ± 0.2 pA/pF for WT (*n* = 4); **Figure 4**]. Tetrodotoxin (TTX), an alpha pore blocker, did not modify the gating pore currents induced by the R225P [at +40 mV: 11.8 ± 1.0 pA/pF (*n* = 5) without TTX vs. 10.9 ± 1.1 pA/pF (*n* = 6) with 10 μM TTX] and R814W mutant channels [at +40 mV 3.4 ± 0.4 pA/pF (*n* = 4) without TTX vs. 3.2 ± 0.3 pA/pF (*n* = 3) with 10 μM TTX; **Figure 4B**]. This confirmed that these cation leak currents do not flow through the alpha pore of the channel but rather through an alternative pathway created by the mutations. The gating pores investigated here were activated by membrane depolarizations. The normalized gating pore currents (I/I_max_) showed that the R225P and R814W gating pores have similar voltage dependence (**Figure 4C**).

**FIGURE 5:**
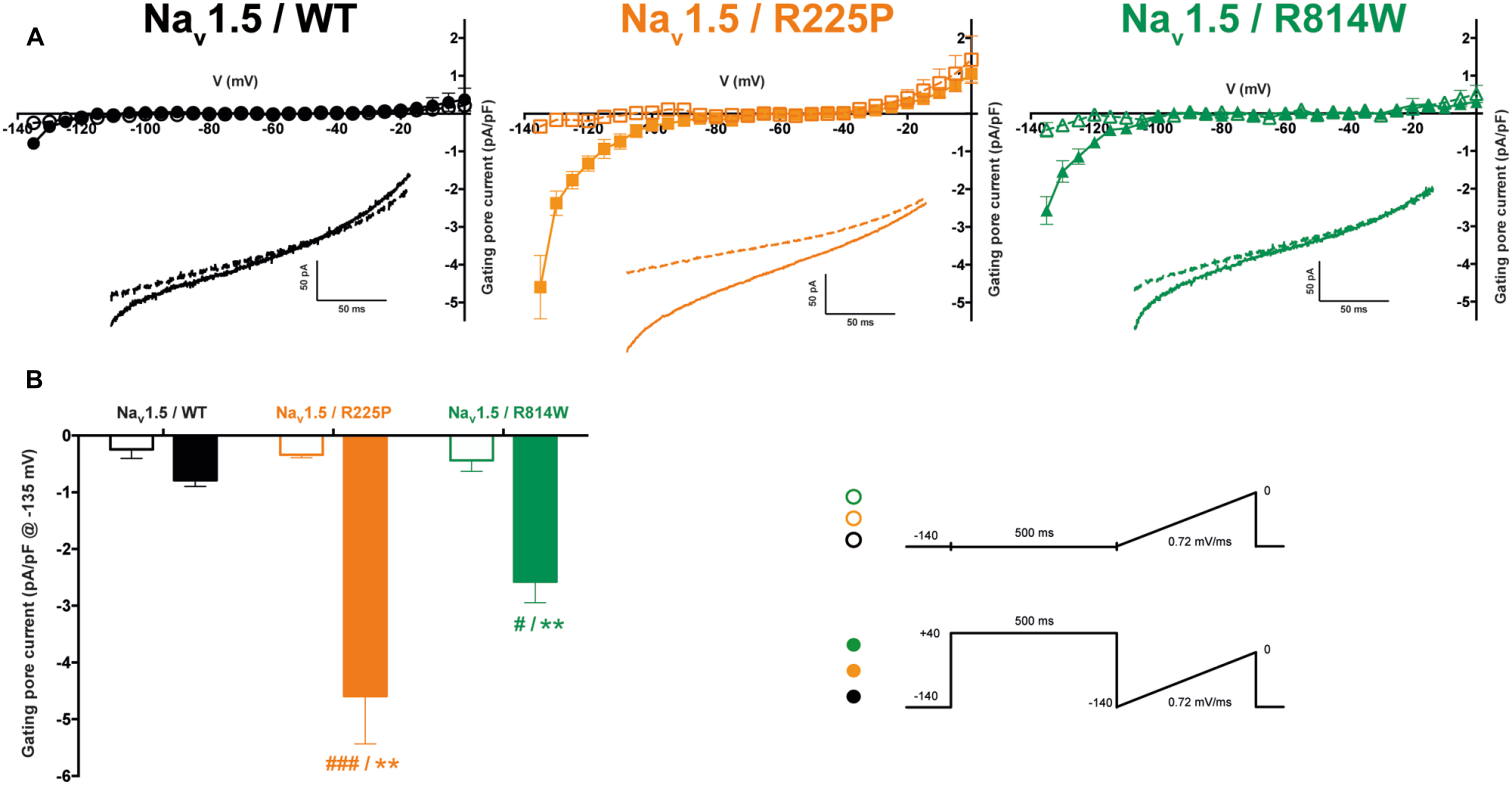
**Gating pore currents after long depolarizations. (A)** Currents generated by ramp pulses (see protocols in inset) for the WT (left), R225P (middle), and R814W (right) channels. The I–V curves were constructed by averaging current values every 5 mV. Voltages were calculated using the known time course of the ramp protocol. A linear leak subtraction around -75 to -45 mV was performed to eliminate the inherent linear leak. The insets show the currents in response to the ramp protocols. Dashed lines indicate the current obtained without pre-depolarization while solid lines indicate the response after a 500-ms pre-depolarization at +40 mV. **(B)** Histogram summarizing the inward gating pore current density at -135 mV recorded with or without a 500-ms pre-depolarization. ^∗^ Indicates a statistical difference between the condition with or without pre-depolarization tested using a *t*-test (^∗∗^*P* < 0.01); # Indicates differences with the WT channel (with the 500-ms pre-depolarization) tested using an ANOVA. (#*P* < 0.05, ###*P* < 0.001) .

Mutations at the bottom of the S4 segment near the cytoplasm amplify the naturally occurring S4 segment immobilization due to long depolarization periods (Sokolov et al., 2008; Fan et al., 2013; Groome et al., 2014; Moreau et al., 2014b, 2015). Gating pore currents were thus recorded (in the presence of 10 μM TTX) using a ramp protocol ranging from -140 to 0 mV at 0.72 mV/ms after a 500-ms pre-depolarization to +40 mV and were compared to those elicited by the same ramp protocol without pre-depolarization (see protocol shown as inset in **Figure 5**). To minimize the impact of capacitance artifacts, gating pore currents were measured from -135 to 0 mV. The I/V curves were constructed by averaging current values every 5 mV. After linear leak subtraction, gating pore currents at hyperpolarized potentials were observed only after a 500-ms pre-depolarization for both mutants [at -135 mV: -4.6 ± 0.8 pA/pF (*n* = 6) vs. -0.3 ± 0.1 pA/pF (*n* = 5) without pre-depolarization for R225P channels and -2.6 ± 0.4 pA/pF (*n* = 4) vs. -0.4 ± 0.2 pA/pF (*n* = 4) for R814W channels]. As expected, no currents were recorded with WT channels (-0.8 ± 0.1 pA/pF, *n* = 5) with a 500-ms pre-depolarization.

## Discussion

Na_v_1.5 mutations are involved in pure electrical disorders such as Brugada syndrome, type 3 long QT syndrome, and cardiac conduction defects with no structural heart diseases (Amin et al., 2010; Moreau et al., 2012, 2013). However, *SCN5A* mutations have been recently associated with cardiac dysfunctions combining arrhythmias and dilatation (McNair et al., 2011; Moreau et al., 2014a). How this gene could be at the origin of a structural heart disease is not fully understood. Gating pores are alternative permeation pathways created in the VSDs of ion channels by the disruption of interactions between the GCTC and the S4 segment (Moreau et al., 2014b). These gating pores notably appear as a result of mutations of the charged residues of the S4 segment. Such pores are known to be permeant to cations with a selectivity sequence that is more permissive for larger ions (Moreau et al., 2014a,b, 2015).

Several mutations on the S4 segment of the Na_v_1.5 channel have been studied due to their association with the pathological development of arrhythmias and dilated cardiomyopathy (Bezzina et al., 2003; Nguyen et al., 2008; Laurent et al., 2012; Mann et al., 2012; Nair et al., 2012; Beckermann et al., 2014; Moreau et al., 2014a, 2015). However, no consensus has emerged on the pathological process leading to this atypical clinical phenotype. In addition, some mutations with similar clinical phenotypes cause different biophysical defects (gain or loss of function; Nguyen et al., 2008; Gosselin-Badaroudine et al., 2014; Moreau et al., 2014a, 2015). We propose that gating pore currents may be a common pathological mechanism linking all mutations on the VSDs of the Na_v_1.5 channel (Gosselin-Badaroudine et al., 2012b, 2014; Moreau et al., 2014a, 2015). We previously showed that gating pore currents are generated by three mutations found in unrelated patients (R219H, R222Q, and R225W) that cause complex arrhythmias and dilated cardiomyopathy (Gosselin-Badaroudine et al., 2012b; Moreau et al., 2015). All these mutations are located in the S4 segment of DI of the Na_v_1.5 channel. The creation of gating pores due to mutations in the DII of the Na_v_1.5 channel has never been investigated before. Such investigations remain of critical importance, because the association between a mutation and the creation of a gating pore cannot be only based on its location. Mutations on Na_v_1.4 DI, DII, and DIII VSDs have been shown to cause the creation of a gating pore. Against all expectations, similar mutations on DIV VSD do not create a gating pore. This difference appears to be related to a larger hydrophobic septum in this particular VSD (Gosselin-Badaroudine et al., 2012a). Oppositely, the study of the similar DIV VSD of Ca_v_1.1 channels has clearly shown that mutations in this domain create gating pores (Fan et al., 2013). Nevertheless, given the high sequence homology between Na_v_1.4 and Na_v_1.5, the creation of gating pores due to mutations in Na_v_1.5 DI, DII, or DIII VSD’s is expected. While single mutations on Na_v_1.5 DI, Na_v_1.4 DI, and Na_v_1.4 DII VSDs have been shown to open a gating pore (Gosselin-Badaroudine et al., 2012a,b; Moreau et al., 2015), other studies revealed that multiple mutations are required to create a gating pore in Na_v_1.2 DII or in the *Shaker* VSD (Sokolov et al., 2005; Gamal El-Din et al., 2010). All together, these results clearly demonstrate that caution is warranted to conclude that a mutation creates or not a gating pore based on its location in the VSD.

We report that gating pores are caused by two other recently characterized mutations, R225P and R814W, which are located in DI and DII, respectively, of Na_v_1.5 (**Figure 1**). We previously studied the R222Q and R225W Nav1.5 DI VSD mutations (Moreau et al., 2015). Besides the creation of gating pores due to both mutations, several biophysical defects were also recorded. This study notably reports an increase (R222Q) or a decrease (R225W) in current density and hyperpolarized (R222Q) or depolarized (R225W) shifts in the steady state activation. Thus, biophysical defects of R222Q pointed toward a gain of function while defects of R225W mutants indicated a loss of channel function (Moreau et al., 2015). Unlike the R225W mutation, the R225P and R814W mutations both cause a gain of channel function, although the defect is less pronounced with the R814W mutant. Gating pores created by these mutations are activated by membrane depolarizations. Interestingly, the biophysical defects observed for alpha pore currents should also affect gating pore currents. The left shift of steady state activation should indicate an early movement of the VSD, resulting in an early opening of the gating pore. Nevertheless, the voltage dependence of the activation is a complicated process resulting from both the movement of the VSD and the coupling between the VSD and the pore domain. The correlation between the alpha pore voltage dependence of activation and the movement of a VSD is thus not necessarily direct. Here, both steady state activation curves were left shifted (**Table 1**). This could be due to an earlier movement of the VSD or a tighter coupling between the VSD and the PD. In the case of an earlier movement of the VSD, this would result in an earlier opening of the gating pore. In our previous study of R222Q and R225W, the voltage dependence of R222Q gating pore was slightly left shifted when compared to the voltage dependence of the R225W gating pore (Moreau et al., 2015). This result would indicate that shifts in the voltage dependence of alpha pore currents would be due to differences in the mutants’ VSDs movement. Indeed, the voltage dependence of the alpha pore current of the R222Q mutant was importantly hyperpolarized when compared with the voltage dependence of the alpha pore current of the R225W mutant (Moreau et al., 2015).

The gating pores currents generated by the R225P and R814W mutations were recorded using Cs^+^ due to its higher permeability. However, based on previously reported selectivity sequences for gating pores, K^+^ and Na^+^ are highly expected to permeate this pathological pore and should affect electrical signals and ionic homeostasis (Moreau et al., 2014a,b, 2015). Like other depolarization-activated gating pores (Sokolov et al., 2008; Fan et al., 2013; Groome et al., 2014; Moreau et al., 2015), the gating pores created by the R225P and R814W mutations remained open after long periods of depolarization, probably due to the temporary partial freezing of the S4 segment (Gamal El-Din et al., 2014; Moreau et al., 2014b). Given the physiological ion concentrations on both sides of the membrane and the biophysical properties of these gating pores, the pores should cause outward K^+^ and Na^+^ leaks under depolarized conditions (during the action potential plateau phase) and inward Na^+^ leak at the end of each action potential (Moreau et al., 2014a,b, 2015). Gating pore currents constitute small leak currents opened as soon as the voltage sensor has moved (Moreau et al., 2014a,b, 2015). Considering their small amplitude, their contribution to the pathologic mechanism could thus be questioned. Nevertheless, it is now well-accepted that small late Na_v_1.5 currents can cause the LQT3 syndrome (Ruan et al., 2009; Amin et al., 2010). Although gating pore currents or late currents are of small amplitude, they are opened for long durations. Consequently, their impact would probably be related to the continuous stress imposed to cardiac myocytes. Several pathological consequences of gating pores have been described in the case of Na_v_1.4 mutations associated with the development of hypokalemic periodic paralysis (HypoPP). In this case, an important elevation of the resting membrane potential (V_Rest_) is thought to be the cause of clinical manifestations (Rudel et al., 1984; Jurkat-Rott et al., 2009). Cation leaks due to gating pores usually represent 1–6% of the alpha pore current amplitude (Moreau et al., 2014a,b). Consequently, charges flowing through the gating pores are thought to not be enough to cause this elevation in V_Rest_. This V_Rest_ elevation would most probably be related to the ionic homeostasis imbalance (intracellular Na^+^ and Ca^2+^ overload) caused by the gating pore. The ionic homeostasis imbalance would block ion channels such as K_ir_ channels and thus cause the V_Rest_ elevation (Hughes and Swaminathan, 2008; Struyk and Cannon, 2008; Tricarico and Camerino, 2011; Moreau et al., 2014a). A similar process is thus expected to affect patient’s cardiac myocytes, resulting in a potentially pathological depolarization of their V_Rest_. This ionic homeostasis imbalance could then result in several other pathological consequences such as the block of connexins, impair the excitation-contraction coupling or also the function and the assembly of contractile proteins (Fabiato and Fabiato, 1978; Chandra et al., 1999; Stergiopoulos et al., 1999; Bukauskas et al., 2001; Duffy et al., 2004; Feng et al., 2008; Moreau et al., 2014a). Taken together, these defects could result in the atypical clinical phenotype associating DCM and mixed arrhythmias. Furthermore, the pathological potential of such gating pore currents is strongly strengthened by the association of a similar mutation in Na_v_1.4 (R1135C/H, R3 – S4, DIII) associated with HypoPP phenotype (Groome et al., 2014).

**FIGURE 6:**
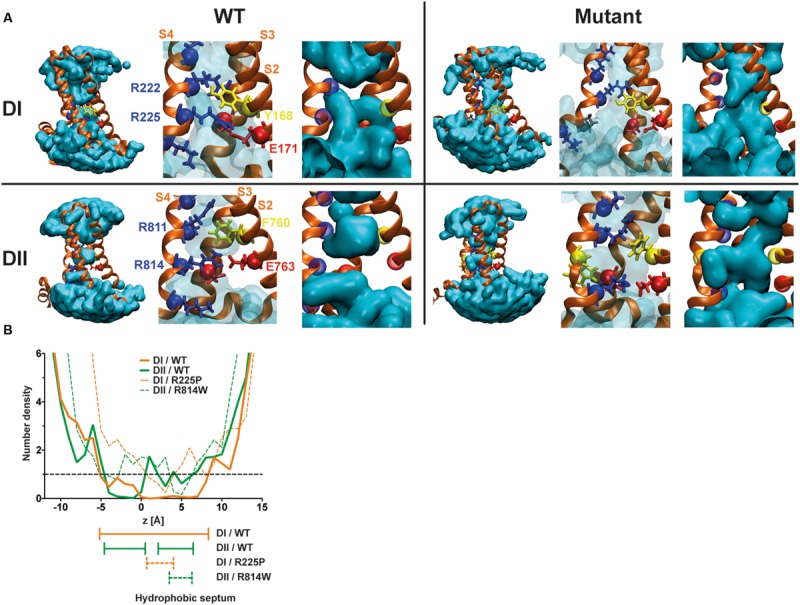
**Molecular basis for the creation of gating pores. (A)** Models of the DI and DII of Na_v_1.5 were built based on previously published results (Moreau et al., 2015). The top row shows the DI models. The bottom row shows the DII models. The left panels represent WT VSDs and the right panels represent mutant VSDs (top R225P, bottom R814W). The protein is shown as orange ribbons, and the water crevice is shown in blue. Positively charged residues in the S4 segment and residues making up the GCTC are highlighted. Positively charged residues are depicted in blue, negatively charged residues in red, aromatic residues in yellow, and the proline in gray. The complete VSD is shown (left) for each panel. A higher magnification of the GCTC is also shown to clearly see the interactions between the S4 segment and the GCTC (middle) as well as the merging of the water crevices from both sides of the membrane (right) for the mutant channels. For purposes of clarity, the S1 segment has been removed. **(B)** Water density profiles of the Na_v_1.5/DI (orange) – DII (green) WT and R225P – R814W mutant channels (dash lines). The histograms were built using a 1-A grid, and the averages were calculated from at least 6 ns of the trajectories. 0 corresponds to the position of the Cα of Y168 of S2 (DI) or the position of the Cα of F760 of S2 (DII).

The R225P and R814W mutations should disrupt interactions between the S4 segment and the GCTC. Based on previously published results (Moreau et al., 2014a,b, 2015), these two mutations should also affect the structure of the VSD, allowing the merging of water crevices as illustrated in **Figure 6**. Once the water crevices merge, a new permeation pathway appears through the usually non-conductive VSD structure, allowing ions to permeate. The substitution of the arginine by a tryptophan (R814W) causes a major impediment to the S4 structure and its movement, resulting in a twist of the segment due to the affinity of tryptophan for lipid tails. The substitution of the arginine by a proline (R225P) should also cause a major modification of the S4 structure. While other reported mutations mainly result in modifications to the side chain of the amino acid, the substitution by a proline affects both the side chain and the alpha carbon. Due to these modifications and based on previously published results, the creation of a continuous water wire through the VSD would thus underlie the creation of gating pores in DI and DII of Na_v_1.5 channels. Indeed, although extensive perturbations to the VSD structure such as the ones possibly induced by the proline and the tryptophan may be hard to resolve in the 100 ns time, our models show that the mutant VSDs are potentially permeable to ions. The water density profile was assessed by counting the average occupancy of water molecules in the VSD along the z-axis (perpendicular to the membrane) over 6 ns (**Figure 6B**). For both mutants the hydrophobic septa (volumes that are not accessible to water) are drastically shorter and more hydrated than the corresponding WT VSDs. In contrast with our previous study (Moreau et al., 2015), along very few angstroms, the water accessible volume in the R225P and R814W VSDs is below one water molecule but clearly above 0. These results more likely indicate that in some frames, no water molecules can be found, while in other frames at the same location a water molecule is found. Since the water density profile is built on at least 600 frames, the mean value is neither 0 nor above 1. This behavior was not observed in WT VSDs as they featured constriction sites in which the water profile value was 0 over several angstroms. Finally, consistently with both our previous study and our experimental data, these results would indicate that R225P and R814W mutated VSDs can conduct ions due to hydrophobic septum disruption resulting in water crevices junction.

Further investigation is also required concerning VSD mutations located outside the S4 segment. Indeed, mutations in S1–S3 segments are known to be associated with the development of arrhythmias and dilated cardiomyopathy (McNair et al., 2004, 2011; Olson et al., 2005; Nguyen et al., 2008; Moreau et al., 2014a; Swan et al., 2014). The creation of a gating pore due to such mutations has never been investigated. Nevertheless, mutations in the VSD but outside the S4 segment might also disrupt interactions between the S4 segment and the GCTC and thus cause the creation of a gating pore.

Gating pores are now well-accepted to be also involved in the pathological development of other muscular or neuronal disorders (periodic paralysis and peripheral nerve hyper-excitability; Sokolov et al., 2007, 2008; Struyk and Cannon, 2007; Miceli et al., 2012; Moreau et al., 2014a). The exact pathological downstream consequences of these cation leaks in myocytes require further exploration. Patient specific induced pluripotent stem cells (hiPSC) or transgenic mouse models could thus constitute models of choice to further investigate the pathologic potential of these leak currents and clearly study their pathologic importance. Nevertheless, we hypothesize that such leaks unbalance ionic homeostasis, thus impairing electrical signals, cellular communication, and excitation-contraction coupling (Moreau et al., 2014a, 2015). The combination of gating pores and defects of the alpha pore could thus lead to the atypical clinical phenotype observed.

## Conclusion

Our study identified gating pores induced by two unrelated Na_v_1.5 mutations. This is the first time that a mutation generating a gating pore outside the DI of Na_v_1.5 has been investigated. The R225P and R814W mutations both caused an atypical clinical phenotype associating cardiac arrhythmia with dilatation, bringing the number of known mutations that create gating pores related to this clinical phenotype to five. Given that the alpha pores of several mutated channels are also defective, gating pores likely contribute to the pathogenesis rather than cause it. However, the presence of these gating pores may determine whether the mutations result in a pure electrical disorder or a pathology associating electrical and morphological abnormalities. We thus propose that gating pores that are created by Na_v_1.5 mutations on the VSD and that are associated with combined electrical and morphological cardiac pathologies should be investigated as a biophysical defect. Finally, while this biophysical characterization is a prerequisite to the better understanding of the pathogenesis, future studies of transgenic animals are warranted for the comprehension of the link between gating pore current and the clinical phenotype combining complex arrhythmias and DCM.

## Author Contributions

AM performed all the experiments involving the biophysical characterization of the wild-type and mutant channels in mammalian cells, he was involved in the recording of gating pore currents, analyzed the data, and helped drafted the manuscript. PG-B performed dynamic simulations and helped drafted the manuscript. MB discuss the data and helped drafted the manuscript MC developed the concept, designed the project and experiments, analyzed and interpreted the results, and drafted the manuscript.

## Conflict of Interest Statement

The authors declare that the research was conducted in the absence of any commercial or financial relationships that could be construed as a potential conflict of interest.
